# *In Utero* Exposure to Maternal Tobacco Smoke and Subsequent Obesity, Hypertension, and Gestational Diabetes Among Women in The MoBa Cohort

**DOI:** 10.1289/ehp.1103789

**Published:** 2011-11-29

**Authors:** Lea A. Cupul-Uicab, Rolv Skjaerven, Kjell Haug, Kari K. Melve, Stephanie M. Engel, Matthew P. Longnecker

**Affiliations:** 1 Epidemiology Branch, National Institute of Environmental Health Sciences, National Institutes of Health, Department of Health and Human Services, Research Triangle Park, North Carolina, USA; 2 Department of Public Health and Primary Health Care, University of Bergen, Bergen, Norway; 3 Medical Birth Registry of Norway, the Norwegian Institute of Public Health, Bergen, Norway; 4 Department of Epidemiology, University of North Carolina Gillings School of Global Public Health, Chapel Hill, North Carolina, USA

**Keywords:** diabetes mellitus, gestational diabetes, hypertension, *in utero*, maternal smoking, MoBa, obesity, tobacco smoke

## Abstract

Background: Environmental factors influencing the developmental origins of health and disease need to be identified and investigated. *In utero* exposure to tobacco smoke has been associated with obesity and a small increase in blood pressure in children; however, whether there is a corresponding increased risk of conditions such as diabetes and hypertension during adulthood remains unclear.

Objective: Our goal was to assess the association of self-reported *in utero* exposure to tobacco smoke with the prevalence of obesity, hypertension, type 2 diabetes mellitus (T2DM), and gestational diabetes mellitus (GDM) in women 14–47 years of age.

Methods: We conducted a cross-sectional analysis of the Norwegian Mother and Child Cohort Study, which enrolled pregnant women in Norway from 1999 thorough 2008. Exposure to tobacco smoke *in utero* (yes vs. no) was ascertained on the baseline questionnaire (obtained at ~ 17 weeks’ gestation); the outcomes were ascertained from the Medical Birth Registry of Norway and the questionnaire. Our analysis included 74,023 women.

Results: Women exposed to tobacco smoke *in utero* had 1.53 times the odds of obesity [95% confidence interval (CI): 1.45, 1.61] relative to those unexposed, after adjusting for age, education, and personal smoking. After further adjustment for body mass index, the odds ratio for hypertension was 1.68 (95% CI: 1.19, 2.39); for T2DM 1.14 (95% CI: 0.79, 1.65); and for GDM 1.32 (95% CI: 1.10, 1.58) among exposed compared with unexposed.

Conclusions: Exposure to tobacco smoke *in utero* was associated with obesity, hypertension, and GDM in adult women. The possibility that the associations were attributable to unmeasured confounding cannot be excluded.

Environmental factors influencing the developmental origins of health and disease need to be identified and investigated (Swanson et al. 2009). To this end, characterizing the relation of *in utero* exposure to tobacco smoke to subsequent health outcomes will serve as an important benchmark. Maternal smoking during pregnancy has a number of well-known short-term adverse effects on offspring, such as shortened gestation and fetal growth restriction [Centers for Disease Control and Prevention (CDC) 2002]. Recent findings suggest that exposure to maternal smoking may also have long-term health consequences. Exposure to tobacco smoke *in utero* has been associated in infants with alterations of blood pressure control mechanisms (Cohen et al. 2010), and in children with an increase in blood pressure (BP) (Brion et al. 2008) and with overweight and obesity (Ino 2010; Oken et al. 2008). During adulthood, the association of obesity with *in utero* exposure to tobacco smoke persists (Power and Jefferis 2002); however, it is not clear whether such exposure is associated with adult diabetes or hypertension (Power et al. 2010; Thomas et al. 2007). Among adults, other adverse outcomes such as carotid wall thickening (Geerts et al. 2008) and raised cholesterol (Jaddoe et al. 2008) have also been linked to *in utero* exposure to tobacco smoke.

Recent evidence suggests that alterations in fetal programming, in response to an adverse fetal environment, might be involved in the origins of chronic conditions later in life (Gluckman and Hanson 2004). In addition, experiments conducted in rodents support an association between fetal nicotine exposure and adverse effects in the offspring such as obesity, increased blood pressure, and altered glucose homeostasis (Bruin et al. 2010). Nevertheless, epidemiologic studies evaluating analogous outcomes during adulthood in relation to *in utero* tobacco smoke are scarce.

We evaluated the association of self-reported *in utero* exposure to maternal tobacco smoke (i.e., exposure 14–47 years ago for women enrolled in the present study) with the prevalence of prepregnancy obesity, hypertension, and type 2 diabetes mellitus (T2DM) in a cohort of pregnant women. We also evaluated the association of self-reported *in utero* exposure to tobacco smoke with prevalence of gestational diabetes mellitus (GDM). GDM and T2DM have somewhat similar pathophysiologies (Buchanan et al. 2007), and women with GDM are at increased risk of developing T2DM (Kim et al. 2002). To our knowledge, GDM has not been previously examined in relation to *in utero* tobacco smoke.

## Materials and Methods

This study was based on the Norwegian Mother and Child Cohort Study (MoBa) conducted by the Norwegian Institute of Public Health (Magnus et al. 2006). MoBa is a cohort based on 108,000 pregnancies from 90,700 women enrolled from 1999 through 2008. Some women in the cohort participated with more than one pregnancy. Most pregnant women in Norway were invited to participate, and 38.5% of invited women participated in the study. Participants were recruited with a mailed invitation before a routine ultrasound examination offered to all pregnant women in Norway at 17–18 weeks of gestation [Norwegian Institute of Public Health (NIPH) 2007]. The study was approved by the Regional Committee for Medical Research Ethics and the Norwegian Data Inspectorate. Informed consent was obtained from each participant. At enrollment (~ 17 weeks of gestation), participants completed a questionnaire about demographics, reproductive health, disease and medication history, and lifestyle. We conducted a cross-sectional analysis based on version 5.0 of the quality-assured data files released for research in July 2010. Our analysis was limited to the woman’s first pregnancy in MoBa.

**Figure 1:**
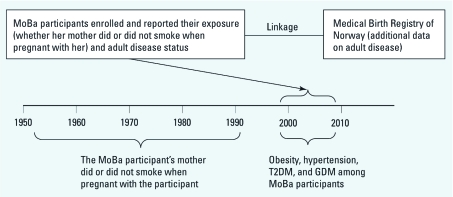
Ascertainment of the exposure and outcomes among women from the MoBa cohort. For example, women born in 1970 who enrolled in MoBa in 2000 when pregnant (at ~ 17 weeks’ gestation), reported their exposure to tobacco smoke when they were *in utero* 30 years previously. Their outcomes in adulthood were reported at enrollment or were ascertained from the MBRN.

*Exposure assessment.* The woman’s own exposure to tobacco smoke *in utero* was ascertained on the baseline questionnaire (Figure 1). Women were asked “Did your mother smoke when she was pregnant with you?” Those who answered “yes” were classified as having been exposed to tobacco smoke *in utero*; those who responded “no” were considered unexposed. For women with more than one pregnancy in the cohort, the consistency of answers across pregnancies was verified. In general, if the woman gave two different answers in two consecutive pregnancies (e.g. yes/no; no/yes; no/don’t know; yes/don’t know), the response was considered inconsistent and the subject was excluded from the analysis. However, if the first answer was “don’t know” and then she gave a different answer after that (yes or no), we used the latter under the assumption that the woman had asked her mother about her exposure *in utero*. The reported *in utero* exposure to tobacco smoke has been shown to be a valid and reproducible measure in the MoBa cohort (Cupul-Uicab et al. 2011a, 2011b). Women were not asked about childhood cigarette smoke exposure.

*Outcomes.* Obesity, hypertension, and T2DM before pregnancy, and GDM during the MoBa index pregnancy (i.e., the woman’s first pregnancy in MoBa) were the main outcomes. Obesity was defined as a prepregnancy body mass index (BMI) ≥ 30 kg/m^2^ (National Institutes of Health 1998). Height and prepregnancy weight were reported by the women on the baseline questionnaire. Women were classified as hypertensive if they met all three of the following criteria: *a*) reported having high blood pressure before pregnancy on the baseline questionnaire (“Do you have or have you had high blood pressure before pregnancy?”); *b*) reported the use of antihypertensive medication before pregnancy on the baseline questionnaire (“If you have taken medication in conjunction with the high blood pressure give the names and when you took them”); and *c*) chronic hypertension was also reported in the Medical Birth Registry of Norway (MBRN; Bergen, Norway). The MBRN is a population-based registry that contains information on all births in Norway since 1967; its data are collected on standard antenatal forms completed by midwives or doctors. GDM and (pregestational) T2DM were ascertained from the MBRN because the questions asked in the baseline questionnaire did not differentiate between types of diabetes. To support the accuracy of the registry’s assessment of the GDM case status, we conducted a validation substudy. Based on the MBRN records, we selected 60 women who had GDM and 79 women who did not, and examined their pregnancy medical records to verify the MBRN classification.

**Figure 2:**
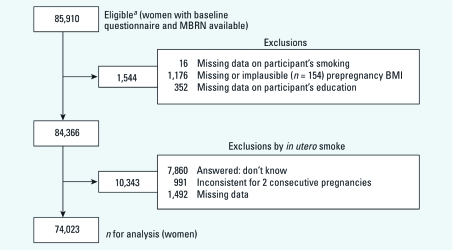
Selection of sample for analysis of *in utero* exposure to maternal tobacco smoke and subsequent obesity, hypertension, and gestational diabetes among women in the MoBa cohort. ***^a^***Based on data set version 5.0.

*Statistical analysis.* For the present analysis we considered as potentially eligible all women with baseline questionnaire and MBRN data for the first pregnancy in which they were enrolled in MoBa (*n* = 85,910). Those missing data on personal smoking, prepregnancy BMI, or education were also excluded (*n* = 1,544). In addition, women were excluded if, for *in utero* tobacco smoke, there were missing data, unknown exposure, or inconsistent answers (*n* = 10,343). After all the exclusions, 74,023 women were included in the analysis (Figure 2).

The associations of *in utero* exposure to tobacco smoke with obesity, hypertension, T2DM, and GDM were assessed separately with logistic regression. Women classified by the MBRN with unspecified diabetes before or during pregnancy were excluded from the analysis of T2DM and GDM. Women with GDM were excluded from all T2DM analysis, and those with T2DM were excluded from all GDM analysis. In addition, women were excluded from the GDM analysis if they reported the use of any diabetic treatment before pregnancy (*n* = 57) or delivered at ≤ 20 weeks of gestation (*n* = 122). All models included *in utero* tobacco smoke as the main exposure and were adjusted for woman’s age, education, and personal smoking as *a priori* selected variables. Additional variables that were associated with any of the outcomes of interest in bivariate analyses (*p* ≤ 0.20) were assessed as potential confounders using the change in estimate method, starting with all variables in the models with deletion of one by one in a stepwise manner (Greenland 1989). None of the tested variables [i.e., parity, physical activity (times/week), and alcohol consumption (drinks/week)] caused ≥ 10% change in the odds ratio (OR) for *in utero* smoking in any of the models. Income was not included as a confounder because of its correlation with education (Spearman *r* = 0.44, *p* < 0.01) and because education was a stronger predictor of the exposure than income. Similarly, year of birth was not added to the models because of its high correlation with age (Pearson *r* = –0.90, *p* < 0.01). Additional adjustment for income and year of birth was done in a sensitivity analysis. BMI is a known risk factor for hypertension, T2DM, and GDM (Li et al. 2007; Vazquez et al. 2007; Yeung et al. 2010); therefore we present the adjusted ORs before and after including BMI in the models. All analyses were done using Stata (release 10.1; StataCorp, College Station, TX, USA).

Birth weight was considered an intermediate variable [exposure to *in utero* tobacco smoke reduces birth weight (Kramer 1987)]; thus, birth weight was included in the models only in a sensitivity analysis. The latter analysis was conducted among the subset of women born in 1967 (when the MBRN was established) or later, with available birth weight (88% of 70,539).

Because the outcomes of interest were correlated, we conducted a number of sensitivity analyses to evaluate whether the associations seen with *in utero* tobacco smoke were independent. Women with T2DM were excluded from the model of obesity. Women with T2DM or heart disease before pregnancy were excluded from the model of hypertension. Women with hypertension, with medication for high cholesterol, or with GDM in a previous pregnancy were excluded from the model of T2DM. Women with hypertension or GDM in a previous pregnancy were excluded from the model of GDM. Excluding women with nonconfirmed hypertension [reported only in the baseline questionnaire or MBRN (*n* = 351)] did not materially change the association with hypertension, so they were included in the analyses. Similarly, excluding women with type 1 DM (*n* = 280) did not change the associations with T2DM and GDM, so they were included in the analyses. In additional sensitivity analyses, women with inconsistent answers or unknown exposure to *in utero* tobacco smoke were included in the models as a third exposure category because they were likely a mix of exposed and unexposed subjects.

Only one subject had obesity, hypertension, and T2DM before pregnancy; 91 had two of these conditions. Therefore, we did a separate analysis to estimate the OR of having at least two of these outcomes versus none. Because women exposed to *in utero* tobacco smoke were more likely to smoke as adults, and smokers tend to be different from nonsmokers regarding socioeconomic and other risk factors (data not shown), multiplicative interactions between *in utero* tobacco smoke (yes/no) and personal smoking (yes/no) were examined in all the models. Interactions between the exposure (yes/no) and BMI (continuous) were also tested in the models for hypertension, T2DM, and GDM. An interaction was considered important if the *p*-value for the interaction term was ≤ 0.10.

## Results

The prevalence of *in utero* exposure to tobacco smoke varied across categories of all variables listed in Table 1 (*p* < 0.05), except for the use of medication for high cholesterol, heart disease, and T2DM before pregnancy (nonsignificant). The highest percentages of *in utero* exposure to tobacco smoke (> 40%) were in women < 20 years of age, those with less than a high school education, those who smoked ≥ 10 cigarettes/day in the 3 months before the index pregnancy, and those with hypertension before pregnancy. The highest prevalence of obesity (≥ 23%) was in women who had hypertension, T2DM, or GDM (Table 1). The most frequent outcome was obesity (9.4%); lower prevalence was observed for hypertension (0.2%), T2DM (0.2%), and GDM (0.7%) (Table 1). The participants’ mean age (± SD) was 30 ± 4.7 years (range, 14–47 years).

**Table 1:** Characteristics of the women according to their *in utero* exposure to tobacco smoke and BMI.

Total	Exposed to tobacco smoke *in utero* [%]	Prepregnancy obesity*^a^* (%)
haracteristic	*n* [%]
*p*-Values from Pearson’s chi-square or Fisher’s exact test were ≤ 0.01 for all variables listed, except for “Use medication for high cholesterol” (*p* = 0.17) and “T2DM” (*p* = 0.07) for *in utero* smoking and “Heart disease” (*p* = 0.25 for *in utero* smoking; *p* = 0.39 for obesity). **a**Prepregnancy BMI ≥ 30 kg/m^2^. **b**Reflects the woman’s exposure 3 months before pregnancy. **c**One unit of alcohol is equivalent to 1.5 cL pure alcohol. **d**Woman’s condition before pregnancy.

Among women with a diagnosis of GDM in the MBRN, 88.3% were validated against the pregnancy medical records; and among those without a diagnosis of GDM in the MBRN record, none was classified with GDM by the medical records.

As expected, women with hypertension, T2DM, or GDM had higher BMIs compared with noncases (Table 2). Compared with unexposed women, those exposed to *in utero* tobacco smoke were more likely, before the pregnancy, to have had obesity [adjusted OR (aOR) = 1.53; 95% confidence interval (CI): 1.45, 1.61], hypertension (aOR = 1.97; 95% CI: 1.39, 2.80), and T2DM (aOR = 1.33; 95% CI: 0.92, 1.93), though the latter did not reach statistical significance. Exposed women were also more likely to develop GDM in the MoBa pregnancy (aOR = 1.52; 95% CI: 1.28, 1.82) (Table 2). ORs adjusted for age only were 0–10% higher than unadjusted estimates (data not shown). After further adjustment for BMI, ORs for hypertension, T2DM, and GDM were attenuated (Table 2); however, the associations of *in utero* exposure to tobacco smoke with hypertension and GDM were still evident.

**Table 2:** ORs for obesity, hypertension, T2DM, and GDM by *in utero* exposure to tobacco smoke among adult women.

Noncases*a*	Cases*a*	Unadjusted	Adjusted*b* (no BMI)	Adjusted*b *(with BMI)
Outcomes	BMI Median (IQR)	BMI Median (IQR)	OR (95% CI)	OR (95% CI)	OR (95% CI)
Before pregnancy										
Obesity		22.7 (3.9)		32.7 (4.1)		1.72 (1.63, 1.81)		1.53 (1.45, 1.61)		
Hypertension		23.0 (4.7)		26.3 (9.2)		1.87 (1.33, 2.64)		1.97 (1.39, 2.80)		1.68 (1.19, 2.39)
Type 2 DM		23.0 (4.7)		27.5 (8.5)		1.39 (0.97, 2.00)		1.33 (0.92, 1.93)		1.14 (0.79, 1.65)
Gestational DM		23.0 (4.7)		27.1 (7.6)		1.60 (1.35, 1.90)		1.52 (1.28, 1.82)		1.32 (1.10, 1.58)
IQR, interquartile range. **a**Numbers of noncases and cases: obesity, 67,071 and 6,952; hypertension, 73,887 and 136; T2DM, 73,249 and 132; GDM, 73,105 and 547. **b**Adjusted for woman’s age, education, and number of cigarettes smoked before pregnancy; the GDM model was adjusted for smoking during pregnancy										

Among women with available birth weight (Table 3), the association of *in utero* smoking with obesity, hypertension, and GDM remained before and after adjusting for birth weight and gestational age. The associations were similar when we restricted the analysis to women born at term (Table 3).

**Table 3:** aORs*^a^* for obesity, hypertension, T2DM, and GDM by *in utero* exposure to tobacco smoke among adult women with available birth weight.

All births (*n* = 61,914)					Term births*b* (*n* = 59,477)		
Before adjusting for birth weight	Adjusted for birth weight*c*	Adjusted for birth weight
Outcomes	Cases	aOR (95% CI)	aOR (95% CI)	Cases	aOR (95% CI)
Before pregnancy										
Obesity		5,927		1.51 (1.43, 1.60)		1.59 (1.50, 1.69)		5,656		1.60 (1.51, 1.70)
Hypertension		104		1.74 (1.17, 2.59)		1.67 (1.11, 2.51)		102		1.67 (1.10, 2.52)
T2DM		94		1.36 (0.89, 2.08)		1.17 (0.76, 1.80)		90		1.13 (0.72, 1.76)
GDM		428		1.49 (1.22, 1.81)		1.39 (1.14, 1.71)		403		1.38 (1.12, 1.70)
**a**Adjusted for woman’s age, education, and number of cigarettes smoked before pregnancy. The GDM model was adjusted for smoking during pregnancy. All models except for obesity were also adjusted for prepregnancy BMI (kg/m^2^). **b**Gestational age ≥ 37 weeks at birth. **c**Additionally adjusted for participant’s gestational age at birth.										

The results from Table 2 remained essentially the same after additional adjustment for income and year of birth; after excluding women with T2DM from the final model of obesity; after excluding women with T2DM or heart disease before pregnancy from the final model of hypertension (with BMI); after excluding women with hypertension, those with medication for high cholesterol, or GDM in a previous pregnancy (*n* = 356) from the final model of T2DM (with BMI); and after excluding women with hypertension from the final model of GDM (with BMI) (data not shown). When women with GDM in a previous pregnancy were excluded from the final model of GDM (with BMI), the adjusted OR for *in utero* smoking was similar (aOR = 1.35; 95% CI: 1.11, 1.64) to the aOR (with BMI) from Table 2. Compared with unexposed women, those with unknown or uncertain exposure to *in utero* tobacco smoke were also more likely to be obese (aOR = 1.33; 95% CI: 1.24, 1.43) and to have hypertension (aOR with BMI = 1.79; 95% CI: 1.14, 2.80), but were similar with regard to T2DM (aOR with BMI = 1.08; 95% CI: 0.65, 1.81) and GDM (aOR with BMI = 1.10; 95% CI: 0.85, 1.42).

Women exposed to tobacco smoke *in utero* were two times more likely to have at least two of the outcomes (obesity, hypertension, or T2DM) before pregnancy (aOR = 2.13; 95% CI: 1.39, 3.28) compared with nonexposed; adjusting for birth weight and gestational age gave similar results (aOR = 2.31; 95% CI: 1.40, 3.82). A statistically significant interaction between *in utero* exposure to tobacco smoke and adult smoking was observed only in the model for obesity (Table 4). *In utero* tobacco smoke was a stronger predictor of obesity among women who were nonsmokers (aOR = 1.75; 95% CI: 1.64, 1.87) as adults than among smokers (aOR = 1.29; 95% CI: 1.18, 1.40; *p*-interaction < 0.01). We also observed stronger associations of *in utero* exposure to tobacco smoke with hypertension, T2DM, and GDM among smokers than among nonsmokers. Interactions between *in utero* smoking and BMI were not observed for hypertension, T2DM, and GDM (*p*-interaction > 0.30).

**Table 4:** aORs*^a^* for obesity, hypertension, T2DM, and GDM by *in utero* exposure to tobacco smoke among women, stratified by adult smoking.*^b^*

Nonsmokers (*n* = 51,852)			Smokers (*n* = 22,171)		
Outcomes	Cases	aOR (95% CI)	Cases	aOR (95% CI)	*p*-Interaction*c*
Before pregnancy										
Obesity		4,565		1.75 (1.64, 1.87)		2,387		1.29 (1.18, 1.40)		< 0.01
Hypertension		105		1.48 (0.98, 2.23)		31		2.56 (1.23, 5.33)		0.24
T2DM		91		1.02 (0.64, 1.62)		41		1.38 (0.74, 2.56)		0.38
GDM		385		1.25 (1.00, 1.55)		162		1.48 (1.08, 2.02)		0.33
**a**Adjusted for woman’s age and education. All models except for obesity were also adjusted for prepregnancy BMI (kg/m^2^). **b**All models except for GDM were stratified by adult smoking before pregnancy; GDM model was stratified by adult smoking during pregnancy (*n* = 55,402 for nonsmokers and *n* = 18,250 for smokers). **c**Interaction term between *in utero* tobacco smoke (yes/no) and adult smoking (yes/no).										

## Discussion

Women exposed to tobacco smoke *in utero* were more likely as adults to be obese and have hypertension. For T2DM we observed slightly increased odds, however, the results were not statistically significant. The odds of developing GDM in pregnancy were also increased in women exposed to tobacco smoke *in utero*; to our knowledge this association has not been examined previously. Our results were similar after adjusting for birth weight and concurrent BMI.

In animals, fetal nicotine exposure results in increased adiposity and body weight, altered perivascular adipose tissue composition and function, raised blood pressure, elevated fasting serum insulin concentrations, and enhanced insulin response to a glucose challenge (Bruin et al. 2010). These changes may have long-lasting adverse effects in the exposed offspring. The doses of nicotine used in animal experiments results in serum cotinine levels (136 ng/ml) (Bruin et al. 2010) that are comparable to those found among moderate smokers (Eskenazi and Bergmann 1995). In humans, other less studied constituents of tobacco smoke (Bruin et al. 2010) might also contribute to the adverse outcomes observed.

An association of *in utero* exposure to tobacco smoke with subsequent childhood obesity has been consistently observed (Oken et al. 2008). Results from a birth cohort showed that this association became stronger with age (up to 33 years), and seemed to be independent of early-life and other adult confounding factors (Power et al. 2010). Results from other studies, however, raise the possibility that the association may be confounded by social factors (Iliadou et al. 2010; Leary et al. 2006).

A previous meta-analysis showed a small increase in BP (adjusted β = 0.62 mmHg; 95% CI: 0.19, 1.05) among children and adolescents exposed to tobacco smoke *in utero* (Brion et al. 2008). However, among adults (~ 45 years of age) this finding tended to be null after accounting for life-time confounding factors (Power et al. 2010).

In studies of *in utero* tobacco smoke and T2DM or elevated percentage of glycosylated hemoglobin, the association was gone after accounting for adult adiposity and other life-time confounding factors (Power et al. 2010; Thomas et al. 2007). Similarly, our results suggested an association between *in utero* tobacco smoke and T2DM; we also observed an attenuation of the OR after adjusting for adult BMI.

*In utero* tobacco smoke was not associated with cardiovascular risk factors other than obesity in a recent study (Power et al. 2010). However, in another study parental smoking during childhood (a surrogate for *in utero* exposure), was associated with a higher prevalence of metabolic syndrome (Hunt et al. 2006). [Metabolic syndrome is diagnosed when at least three of the following conditions are present: central obesity, elevated triglycerides, reduced high-density lipoprotein cholesterol, high blood pressure, and elevated fasting glucose (Alberti et al. 2009).] We did not evaluate metabolic syndrome as an outcome, although women exposed to tobacco smoke *in utero* were more likely to have at least two of the prepregnancy outcomes studied. Obesity, hypertension, and T2DM are chronic conditions that become more prevalent at older ages (Deshpande et al. 2008; Ong et al. 2007; Pi-Sunyer 2002); as this cohort ages, the associations we observed may be estimated more precisely, perhaps becoming easier to detect as more women develop the outcomes of interest. The association of *in utero* exposure to tobacco smoke with GDM may also have implications for future evaluations of the association between *in utero* exposure to tobacco smoke and T2DM (GDM is a risk factor for T2DM).

The prevalence of obesity and T2DM tends to be slightly higher in women than in men, while the prevalence of hypertension in early adulthood tends to be higher in men than in women (American Diabetes Association 2010; Chobanian et al. 2003; Pi-Sunyer 2002). Differences between the sexes were not reported in most previous studies of *in utero* exposure to tobacco smoke (Hunt et al. 2006; Power et al. 2010; Thomas et al. 2007). The association of *in utero* exposure to tobacco smoke with obesity was, however, slightly stronger among men in one study (Power and Jefferis 2002). Thus, our results may not be generalizable to males.

In the present study and previous studies, many factors (e.g., social, demographic, and lifestyle) were associated with *in utero* tobacco smoke; thus, confounding by unmeasured factors may explain some or all of the associations we identified (Donovan and Susser 2011). And many examples exist where once confounding by lifetime factors is accounted for, the relationship weakens. However, adjustment for risk factors that are affected by the exposure (i.e., birth weight and BMI) is controversial because standard methods used to assess the direct effect of an exposure on a given outcome after controlling for an intermediate variable may result in bias (Cole and Hernan 2002). In some studies, father’s smoking is as strongly associated with total fat mass and blood pressure in children as is mother’s smoking, which has been interpreted as evidence of confounding (Brion et al. 2007; Leary et al. 2006). However, among pregnant women exposed to secondhand tobacco smoke, levels of cotinine (the primary metabolite of nicotine) in fetal fluids can be higher than those found in maternal serum (Jauniaux et al. 1999). Thus, *in utero* exposure to tobacco smoke from the partner may truly have an impact on the fetus.

The estimation of a controlled direct effect of *in utero* exposure to tobacco smoke on the outcomes (i.e., obesity, hypertension, T2DM, and GDM) would have required the use of causal methods and assumptions that do not necessary hold in the present setting (Cole and Hernan 2002); therefore, we used a traditional approach. In our analyses adjusted for potential intermediate variables (e.g., BMI), we are implicitly assuming that there are no unmeasured common causes of the intermediate variable and the outcomes (i.e., hypertension, T2DM, and GDM), which is also a questionable assumption.

Our stratified analysis showed somewhat stronger associations of *in utero* exposure to tobacco smoke with hypertension, T2DM, and GDM among smokers than among nonsmokers, but a stronger association with obesity among nonsmokers than among smokers. A potential explanation for the weaker association with obesity among smokers is that women who smoke tend to gain less weight as they age than do nonsmokers (CDC 2002) or perhaps because the “effect” of *in utero* exposure to tobacco smoke is additive (i.e., smokers may have other adverse exposures or behaviors related to their smoking and positively associated with obesity, thus *in utero* smoking has little “effect” on their outcome).

Our results were derived from a cross-sectional analysis of self-reported early-life exposure to tobacco smoke and adult adverse outcomes. Women were unaware of the study hypothesis, so differential reporting of outcomes by exposure status seems unlikely.

Women reported their own *in utero* exposure to tobacco smoke 14–47 years later. Random errors in the classification of the exposure might have occurred, causing an underestimation of the ORs. The reported exposure to maternal tobacco smoke *in utero* by the adult offspring has been shown to be valid and reproducible (Cupul-Uicab et al. 2011a, 2011b; Simard et al. 2008). In MoBa, the validity of the exposure was assessed indirectly using the birth weights of a subset of participants. In general, maternal smoking during pregnancy has been associated with an average reduction of 149 g in birth weight (Kramer 1987); among MoBa participants the observed reduction was 181 g (Cupul-Uicab et al. 2011b). The reproducibility of self-reported *in utero* exposure to tobacco smoke in MoBa was high (weighted κ = 0.80) (Cupul-Uicab et al. 2011b). The intensity of *in utero* exposure to tobacco smoke was not ascertained in MoBa; therefore we could not evaluate a dose–response relationship. If women who experienced very intense exposure to maternal tobacco smoke *in utero* were underrepresented in our study, we may have underestimated the ORs. Information on *in utero* exposure tobacco smoke by trimester as well as the number of mothers who stopped smoking during pregnancy was not ascertained, limiting our ability to assess critical windows of exposure. We were unable to discriminate between *in utero* tobacco smoke and childhood exposure; however, it is likely that women whose mothers smoked while pregnant with them (i.e., exposed *in utero*) also experienced continuous exposure during childhood (Weinberg et al. 1989). Information on childhood exposure to tobacco smoke from both parents as well as paternal smoking during the mother’s pregnancy was not ascertained in MoBa. Thus, confounding by childhood exposure to tobacco smoke from parents cannot be ruled out.

The outcomes studied were questionnaire-based or ascertained from the MBRN; however, we were able to assess the validity of the GDM within the MoBa cohort. The positive predictive value (88%) of the MBR against medical records in the present study was similar to what was reported previously for the MBRN in 1998 (89%, validated against medical records) before changes were introduced to the MBRN form (Stene et al. 2007). Self-reported height and weight give accurate estimates of BMI among adults (Brunner Huber 2007); and agreement of medical records with self-reported hypertension and diabetes in other populations has been moderate to high (Okura et al. 2004). In addition, known predictors of the outcomes were confirmed in the present study. For example, BMI was negatively associated with education (Table 1); hypertension and diabetes (T2DM and GDM) were positively associated with prepregnancy BMI (data not shown). *In utero* exposure to smoking was not associated with type 1 diabetes mellitus (data not shown), further supporting the specificity and validity of the diabetes outcomes. Nonetheless, random errors in the classification of the outcomes may have occurred, leading to an underestimation of the associations.

Women who participated in MoBa are not a representative sample of pregnant women from Norway; the prevalence of exposures and outcomes were biased. For example, the prevalence of smoking at the end of the pregnancy in Norway was 10.8% (MBRN; between 2000 and 2006) and the corresponding prevalence in MoBa was 6.1% (Nilsen et al. 2009). We expected an underestimation of the prevalence of *in utero* exposure to tobacco smoke in our sample because women who smoke as adults were more likely to report *in utero* exposure (data not shown). However, previous analyses suggest that bias in the estimated parameters due to self-selection is negligible when evaluating exposure–outcome associations in the cohort (Nilsen et al. 2009); this tendency may hold when assessing early life exposures in relation to adult outcomes.

## Conclusion

Overall, self-reported *in utero* exposure to tobacco smoke was associated with obesity, hypertension, and GDM in adult women. The possibility that the associations were attributable to unmeasured confounding cannot be excluded.
